# Effect of pollination time, the hour of daytime, pollen storage temperature and duration on pollen viability, germinability, and fruit set of date palm (*Phoenix dactylifera* L.) *cv* "Deglet Nour"

**DOI:** 10.1016/j.sjbs.2021.09.062

**Published:** 2021-10-05

**Authors:** Karim Kadri, Mohammed Elsafy, Souhayla Makhlouf, Mohamed A Awad

**Affiliations:** a Laboratory of biotechnology and oasis genetic resources, Regional Center of Research in the Agricultural Oasis of Degache, BP 62, km1 road of Tozeur, 2260 Degache, Tunisia; b Department of Plant Breeding, Swedish University of Agricultural Sciences, Växtskyddvägen1, P.O. Box 190, SE-234 22 Lomma, Sweden; c Department of Arid land Agriculture, Faculty of Meteorology, Environment and Arid land Agriculture, King Abdulaziz University, P.O.Box. 80208, Jeddah, Saudi Arabia; d Pomology Department, Faculty of Agriculture, Mansoura University, El-Mansoura, Egypt

**Keywords:** Date palm, Pollination, Pollen viability, Pollen germinability, Fruit set, Yield

## Abstract

Success artificial pollination with viable pollen is crucial process in the production chain of date palms. This study evaluated the impact of pollen storage temperature and duration, pollination time following spathe cracking, and the hour of daytime on pollen viability, germinability, fruit set and yield of 'Deglet Nour' date palm cultivar. In *in vitro* tests, fresh pollen showed the maximum viability (96.3%) and germination (85%) but it decreased thereafter upon the storage temperature (28, 4 and −30 °C) and duration (3, 6, 9 and 12 months). In this respect, pollen stored at −30 °C retained highest viability and germinability followed by those stored at 4 and then at 28 °C. In filed experiments, fruit set was 85, 75, 65, and 45% with pollination using fresh pollen, or pollen stored at −30, 4 and 28 °C, respectively. Fruit set was 95%, 75%, and less than 50%, for pollination performed on the same day of spathe cracking, 6 and 12 days later, respectively. The highest fruit set percentage and yield/bunch were obtained with pollination performed between 12.0 pm and 15.0 pm in contrast to 8.0–11.0 am or 16.0–17.0.

## 1 Introduction

Date palm (*Phoenix dactylifera L.*) trees are considered the base of oasis crop systems as a source for staple food production in hyper-arid environments and offering protection for the other underlying crops. Moreover, in some oasis located in the South West of Tunisia, date palm trees play very important role both socially and economically representing the main crop on which the regional economy is based almost entirely (Kadri et al., 2019, Othmani et al., 2020). As traditional symbol of fertility and prosperity of the Saharan and Pre-Saharan zones, the date palm now offers Tunisia a product that makes the fortune of the agricultural sector. Exporting to more than 80 countries, Tunisia is in first place worldwide in terms of commercial transactions of dates, with a market share of around 20% (Kadri et al., 2019, Othmani et al., 2020). Success artificial pollination with viable pollens is crucial process in the production chain of date palms. Storage of pollens with retaining higher viability and germinability is a critical practice for ensuring pollination, maintaining regular bearing of date palms, especially for early season flowering female cultivars and conservation of biodiversity (Shivanna et al., 1991, Mesnoua et al., 2018). With the climatic changes that may cause variation in flowering time among the female trees of several date palm cultivars and their respective suitable male trees, storage of pollen grains become an indispensable practice. Thus, storage conditions and duration for retaining pollen viability and germinability are worthy of research. According to Ibrahim and Kobbia (1998), pollens can be collected at anthesis and stored under dry conditions tightly in a bottle at cold or room temperature for four weeks without loss of viability or pollination efficiency. The viability and germinability of date palm pollen stored under room temperature (25–30 °C) or cold storage at 3–4 °C (Shaheen et al., 1986, Maryam et al., 2015, Mesnoua et al., 2018), freezing at −20 °C and by cryopreservation at −196 °C (Ateyyeh, 2012) were evaluated for various date palm males in different countries. The pollination time following spathe cracking might extend over 40 days and greatly varies up on cultivars and climatic conditions especially temperature (El Fawal, 1972). The receptivity period of North African cultivars greatly varies from one cultivar to another (30 days for 'Bousthami Noire', 7 days for 'Deglet Nour', 8 days for 'Jihel' and 'Ghars' and only 3 days for 'Medjhool', 'Boufeggous' and 'Iklane') (Ream and Furr, 1969, Djerbi, 1995). In addition, the hour of daytime for pollination performance might also affect fruit set percentage. Iqbal et al. (2014) reported that the maximum fruit set and yield in 'Dhakki' date palm cultivar was obtained with pollination being performed between 12.0 pm and 1.0 pm. This study aims to evaluate the impact of pollen storage temperature and duration, pollination time following spathe cracking, and the hour of daytime on pollen viability, germinability, fruit set and yield of 'Deglet Nour' date palm cultivar growing at Degache oasis as an attempt to optimize the pollination process for higher fruit set and yield.

## 2 Materials and methods

### 2.1 Plant materials and pollen storage conditions

The experiments of this study were conducted at the experimental plot of the Regional Center of Research in The Agricultural Oasis of Degache located at the South West of Tunisia during two successive growing seasons of 2018 and 2019. Three female palms of 'Deglet Nour' cultivar of similar age and growth characteristics were selected. A male tree 'P7′ clone belonging to the same experimental plot was selected and used for pollen source. The collected pollen grains were air dried and kept in hermetically sealed glass boxes and stored at either 28 (the yearly mean ambient temperature), 4 or −30 °C for 3, 6, 9 and 12 months. A portion of fresh pollens collected from the same male tree was used as a control. Both female and male trees received the normal regular cultural practices and a crop load of 12 bunches/female palm was adjusted.

### 2.2 In vitro fresh and stored pollen viability and germinability evaluations

#### 2.2.1 In vitro evaluation of fresh and stored pollen viability

The pollens viability evaluation was conducted in triplicate samples by staining the fresh and stored pollens in slide with 1–2 drops of 1% acetocarmine dye with a coverslip and viewing them under a microscope with a magnification of 400 X (Shaheen (2004). The normal appearance of red-stained pollen grains was considered viable while the colorless ones were recorded as non-viable.

#### 2.2.2 In vitro evaluation of fresh and stored pollen germinability

Evaluation of pollens germination was performed in triplicate samples using a standard media consisting of (Ca (NO_3_)2·.2H_2_O (0.20 g), H3BO_3_ (0.20 g), KNO_3_ (0.10 g), MgSO_4_·7H_2_O (0.20 g), agar (10 g) and sugar (150 g) per liter (Brewbaker and Kwack, 1964). The pH of the media was adjusted to 5.7 before agar addition and then sterilized in the autoclave for 20 min. Following that, the media was poured into Petri dishes under a laminar air flow hood and placed at room temperature to settle and cool. The fresh and stored pollens were sown on the dishes using brushes to have well separated pollens and incubated at 30 °C for 24 h. The germination of the fresh and stored pollens was evaluated using a microscope (400X magnification) and an average of five microscopic fields of vision was achieved for each storage temperature. The pollen germination percentage was calculated as follows: Germination (%) = (germinated pollen/total number of grains) × 100.

### 2.3 Field pollination experiments

#### 2.3.1 Effect of pollen storage temperature and duration on fruit set and yield

Four spathes were selected on each of the three female trees during the middle of the flowering period. One bunch on each tree (representing a total of 3 replicates per treatment) was pollinated with either fresh pollens (as a control treatment) or pollens stored for 12 months at each of the respective storage temperature of 28 °C, 4 °C, and −30 °C. Following pollination, all bunches were wrapped with Kraft paper bags for three weeks to avoid contamination from other pollens. Pollination was carried out by inserting small cotton pouche emerged in 0.5 g pollens corresponding to each storage temperature in each bunch. At harvest, the weight of all bunches of each treatment were recorded.

#### 2.3.2 Effect of pollination time following spathe cracking on fruit set and yield

Tow spathes were selected on each of the three separate female trees (representing six replicates per treatment) during the middle of the flowering period. Each spathe was divided into 10 groups (treatments) each of 5 spikelets and each group was then covered with waterproof Kraft paper bag. After every 3 days and until 30 days, the bags opened to manually inversely insert two male spikelets containing fresh pollens (collected 3 days before pollination) and then directly closed again. All bags were removed after 15 days following the last pollination time for fruit set percentage calculation. Total weight per bunch (Kg) was calculated by multiplying the mean fruit weight in each 5 spikelets by 50 as the mean of total number of spikelets in a bunch.

#### 2.3.3 Effect of the hour of daytime pollination on fruit set

Two spathes were selected on each of the three female trees (representing six replicates per treatment) during the middle of the flowering period. Each spathe was divided into 10 groups (treatments) each of 5 spikelets. The pollination started at 8:00 am, and repeated for each group every hour until 17:00 pm using fresh pollens (collected 3 days before pollination). Groups were covered with kraft paper bags for three weeks and then removed for fruit set percentage calculation.

For all the above experiments, following Kraft paper bags removal, the seated fruits and number of dropped flowers scars were counted on each spikelet and fruit set percentage was calculated on a composite sample of 5 spikelets per bunch (Mustafa et al., 2014). Fruit set percentage was calculated at early stage of fruit development; however, the retention percentage was calculated at harvest.

### 2.4 Statistical analysis

The data were statistically analyzed for each experiment as a completely randomized design (CRD) by analysis of variance (ANOVA) using the statistical package software SAS (SAS Institute Inc., 2000, Cary, NC., USA). Comparisons between means were made by the least significant differences (LSD) at *P ≤* 5%.

## 3 Results

### 3.1 In vitro evaluation of fresh and stored pollen viability

**Fig. 1:**
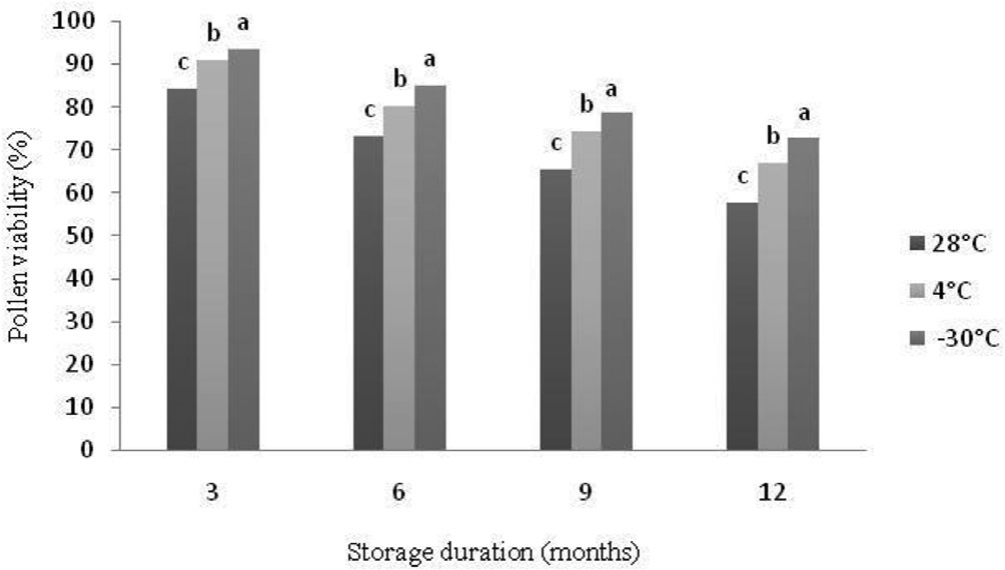
The effect of storage temperature for different durations on *in vitro* pollens viability (%) of ‘Deglet Nour’ date palm cultivar. At each duration, means followed by the same letter are not significantly different at level *P ≤* 0.05 (LSD value = 1.38, 1.08 and 1.22 for 28, 4 and −30 °C, respectively).

**Fig. 2:**
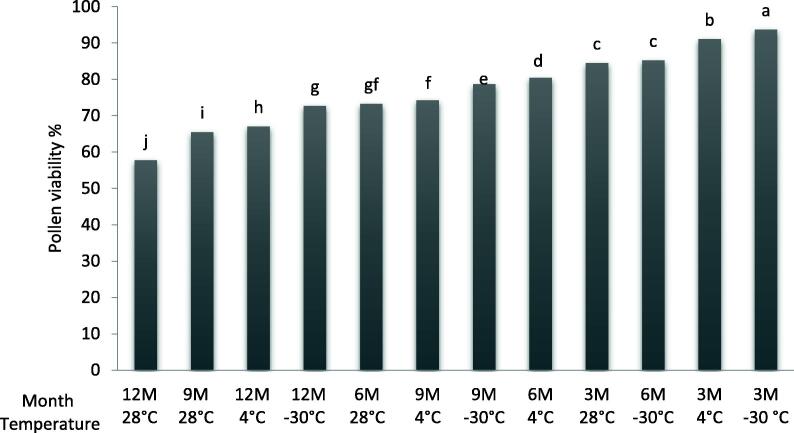
The interaction effect between storage temperature and duration on *in vitro* pollens viability (%) of ‘Deglet Nour’ date palm cultivar. Means followed by the same letter are not significantly different at level *P ≤* 0.05 (LSD value = 1,67).

Pollen stored at low temperature retained their viability better than those stored at room temperature (Fig. 1, Fig. 2; Table 1). The viability of stored pollen at different temperature was almost similar to each other during the first 3 months. However, after long term storage, pollen stored at 28 °C quickly losses their viability compared to those stored at −30 °C and those stored at 4 °C (Fig. 1). Fresh pollen showed the maximum viability (96.3%), but it decreased as the storage time increased to lower level upon on the storage temperature (Fig. 2). After 3 months of storage, frozen pollen exhibited the maximum viability (93.7%), while this value decreased to 84.5% for the pollens stored at 28 °C. After 6 months of storage, pollen viability showed a slight decrease at all storage temperatures with the highest level for frozen ones (85.2%). However, after 12 months of storage, viability percentage varied upon storage temperature with a decrease of about 60% for pollen stored at 28 °C. However, pollen stored at 4 °C and −30 °C retained acceptable viability level varying respectively between 67 and 72% with a decrease of less than 30% compared to initial (Fig. 2).

**Table 1:** Analysis of variance for the effect of temperature and storage duration on pollen viability of ‘Deglet Nour’ date palm cultivar.

Factors	D.F.	MS	*P ≤* 0.05
Duration	3	1557.19031	0.00
Temperature	2	781.036133	0.00
Duration × Temperature	6	7.71017599	4.7586E-07
Error	48	0.78552419	

### 3.2 In vitro evaluation of fresh and stored pollen germinability

**Fig. 3:**
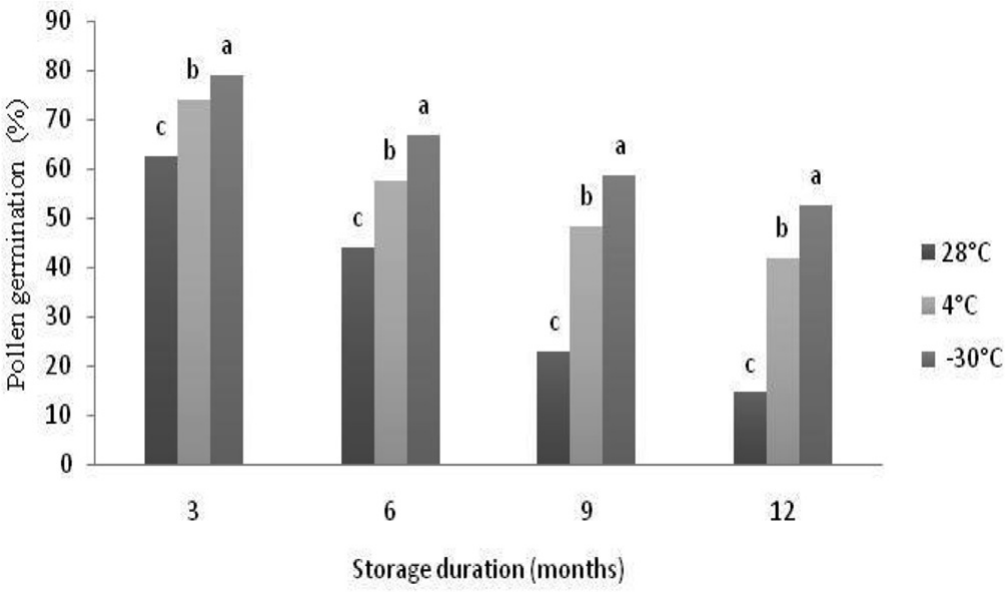
The effect of storage temperature for different durations on *in vitro* pollen germination (%) of ‘Deglet Nour’ date palm cultivar. At each duration, means followed by the same letter are not significantly different at level *P ≤* 0.05. (LSD value = 2.89, 1.91 and 1.90 for 28, 4 and −30 °C, respectively).

**Fig. 4:**
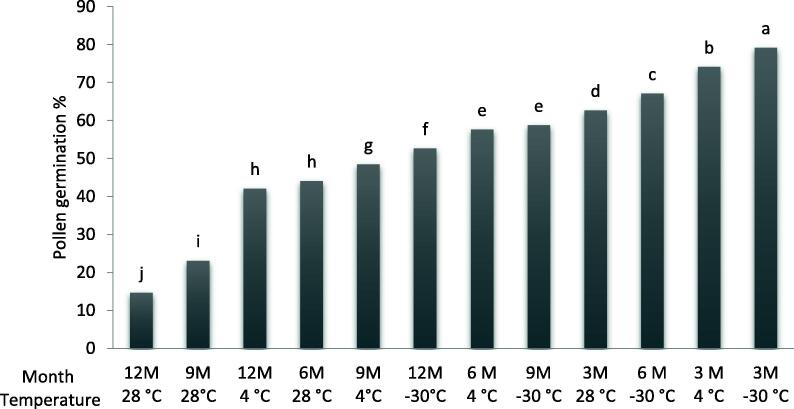
The interaction effect between storage temperature and duration on *in vitro* pollen germination (%) of ‘Deglet Nour’ date palm cultivar. Means followed by the same letter are not significantly different at level *P ≤* 0.05 (LSD value = 2,34).

The germination percentage of pollen stored at different temperatures gradually decreased with the increase of storage duration (Fig. 3; Table 2). Pollen stored at 4 °C retained higher germination percentage compared to those stored at ambient conditions (28 °C). The initial germination percentage of freshly collected pollens was 85.9% while, it decreased to 52.2% at −30 °C after 12 month of storage. The maximum germination percentage was observed after 3 month of storage (79.2%) at −30 °C and the minimum after 12 months (14.7%) at 28 °C. Storage at 28 °C recorded a remarkable decrease in germination percentage ranging from 62.7% after 3 months to 14.7% after 12 months. After 6 months, there was a significant reduction in germination percentage at all storage temperatures compared to fresh pollen, but with different extends (Fig. 4; Table 2). Storage of pollens at 4 °C retained acceptable germination of about 42.1% but with a decrease of almost 50% after 12 months of storage. The highest germination percentage after 12 month of storage was recorded at −30 °C with a mean value of 52.6% and a rate of decrease of 40% (Fig. 4).

**Table 2:** Analysis of variance for the effect of temperature and storage duration on pollen germinability of ‘Deglet Nour’ date palm cultivar.

Factors	D.F.	MS	*P ≤* 0.05
Duration	3	3659.65918	0.00
Temperature	2	4177.94971	0.00
Duration × tempeature	6	147.042221	7.447E-20
Error	48	2.66845083

### 3.3 Effect of pollen storage temperature on fruit set and yield

The highest percentage of fruit setting was recorded for the control treatment (fresh pollen) with an average of 89.7% over 2018 and 2019 seasons giving a final retention percentage of 77.5% and a total bunch weight of 13 kg (Table 3). However, the use of pollen stored at 28 °C resulted in very low fruit set of about 20.3% giving a final bunch weight of less than 2 kg. Pollens stored at 4 °C gave a respectable germination percentage (62.5%), with a bunch weight of 5 kg. In this frame, pollens stored at −30 °C gave germination and viability percentages of 80% and 83%, respectively (Fig. 2, Fig. 3) resulting in a significant fruit setting of up to 68% and a final yield of 8.5 kg per bunch (Table 3). Accordingly, storing pollens at −30 °C was the most effective temperature where, pollens retained acceptable germination and viability percentages up to 12 months.

**Table 3:** Effect of pollens storage temperature for 12 months on fruit set and fruit retention percentage, and yield/bunch of ‘Deglet Nour’ date palm cultivar.

Storage temperature	Fruit set (%)		Fruit retention (%)		Yield/bunch (kg)	
	2018	2019	2018	2019	2018	2019
Fresh pollen	89.80^a^	89.53^a^	76.59^a^	79.16^a^	12.10^a^	14.36^a^
28 °C	22.46^c^	18.16^d^	16.55^d^	12.21^d^	2.26^d^	1.58^d^
4 °C	62.66^b^	63.38^c^	53.32^c^	48.40^c^	4.33^c^	5.22^c^
−30 °C	66.04^b^	68.97^b^	60.58^b^	54.42^b^	6.94^b^	8.52^b^
LSD	3.96	4.31	4.76	6.64	1.15	0.85

Means within each column followed by the same letter are not significantly different at level *P ≤* 0.05.

### 3.4 Effect of pollination time following spathe cracking on fruit set and yield

Fruit set percentage decreased with the delay of pollination time following spathe cracking (Table 4). The maximum fruit set (84.3% and 87.5% in 2018 and 2019, respectively) was recorded by pollination on the same day of spathe cracking and consequently with a maximum yield per bunch (17 Kg). It is noted that flower receptivity remains active producing an acceptable level of fruit set up to the 6th day following spathe cracking with a reduction of about 20% compared to control. However, pollination at the 9th day following spathe cracking exhibited a remarkable reduction in fruit set of up to 50% and consequently a significant decrease in yield up to 60% compared to control. While, pollination after 15 days of spathe cracking gave moderate fruit set below 35% with fruit retention below 30%, and consequently a low yield of about 3 Kg per bunch (Table 4). However, pollination after 3 weeks of spathe cracking, decreased fruit set by more than 75% with an average yield per bunch of less than 1.5 kg. Beyond 4 weeks of spathe cracking, the results showed a remarkable deterioration in fruit set (7%), with a retention of less than 3% and a yield reduction of 98% compared to control. The yield per bunch with pollination performed 30 days after spathe cracking showed a reduction rate of 98% (Table 4).

**Table 4:** Effect of pollination time following spathe cracking on fruit set and fruit retention percentage, and yield/bunch of ‘Deglet Nour’ date palm cultivar.

Days after spathe cracking	Fruit set (%)		Fruit retention (%)		Yield/bunch (kg)	
	2018	2019	2018	2019	2018	2019
0	84.30^a^	87.51^a^	65.93^a^	64.38^a^	17.70^a^	17.25^a^
3	73.87^b^	75.38^b^	56.43^b^	52.38^b^	10.67^b^	10.69^a^
6	67.05^b^	63.53^c^	46.49^c^	48.67^b^	6.92^c^	7.82^c^
9	42.67^c^	45.30^d^	39.75 ^cd^	39.84^c^	5.71^c^	5.17^d^
12	40.50^c^	41.92^de^	33.39^de^	36.90 ^cd^	3.63^d^	4.36^d^
15	35.23 ^cd^	33.51^ef^	29.31^e^	32.73^de^	3.07^d^	3.33^e^
18	30.39^de^	29.10 ^fg^	26.17^e^	28.73^e^	2.30^de^	2.60^ef^
21	24.17^ef^	22.70^gh^	18.05^f^	21.65^f^	1.21^ef^	1.72 ^fg^
24	19.94^f^	17.39 ^h^	16.50 ^fg^	17.20^f^	0.87^gh^	1.03^gh^
27	14.8 ^fg^	12.08^hi^	9.83^gh^	9.57 ^g^	0.33 ^h^	0.46 ^h^
30	7.95 ^g^	5.88^i^	4.00 ^h^	2.85 ^h^	0.10 ^h^	0.11 ^h^
LSD	8.91	9.94	7.59	5.64	1.65	1.04

Means within each column followed by the same letter are not significantly different at level *P ≤* 0.05.

### 3.5 Effect of the hour of daytime pollination on fruit set percentage

**Fig. 5:**
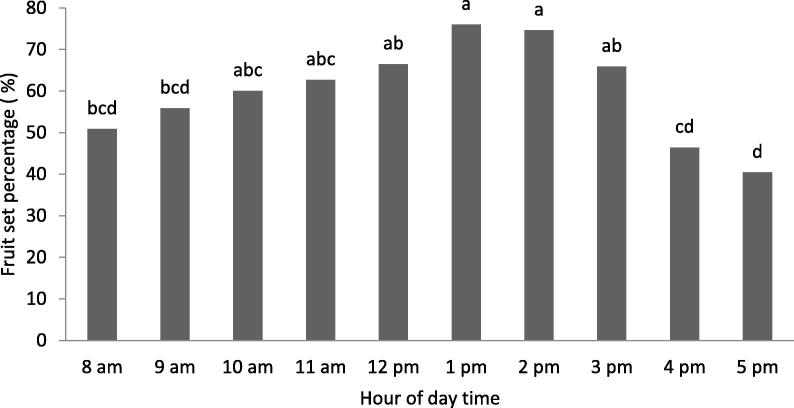
Effect of the hour of daytime pollination on fruit set of ‘Deglet Nour’ date palm cultivar. Means followed by the same letter are not significantly different at level *P ≤* 0.05 (LSD value = 14.29).

The maximum fruit set (75%) was recorded by pollination conducted between 1.0 pm and 2.0 pm (Fig. 5; Table 5). However, the pollination performed between 8.0 and 9.0 am gave fruit set less than 55%. While, the pollinations performed at the end of the day (16.0 and 17.0 pm) gave lower fruit set (below 50%). Pollination carried out in other time intervals during daytime produced acceptable fruit set level varying between 55 and 70% (Fig. 5).

**Table 5:** Analysis of variance for the effect of the hour of daytime pollination on fruit set of ‘Deglet Nour’ date palm cultivar.

Factors	D.F.	MS	*P ≤* 0.05
Time (h)	9	683.7885	7.91E-06
Error	40	101.16405	

## 4 Discussion

According to the obtained results, storage temperature is decisive for date palm pollen viability and germinability. Pollen stored at 28 °C gradually lost their germinability reaching only 20 and 14% after 9 and 12 months, respectively. On the other hand, pollen stored at 4 °C or those frozen at −30 °C showed a slight decrease in their viability and germinability after 6 months of storage. Freezing at −30 °C proved the optimal temperature for keeping high pollen viability (70%) and germinability (60%) after 12 months of storage. Similar results were recently reported in which the maximum *in vitro* pollen germinabilities were obtained with storage at −20 °C followed by 4 °C for several Tunisian (El Kadri and Ben Mimoun, 2020) and Algerian (Mesnoua et al., 2018) male date palm genotypes. Our results confirm previous studies conducted on different date palm male genotypes where the optimum temperature for pollen storage varies between −20 °C to −80 °C that limit the ongoing metabolic activities in pollen (Mortazavi et al., 2010, Maryam et al., 2015, Kadri et al., 2017). Moreover, cryogenic storage (-196 °C) was suggested by Anushma et al. (2018) as a promising method to store date palm pollen for commercial date production, breeding programs and conservation of elite pollen genotypes. In the current study, indeed, frozen date palm pollens exhibited higher viability than those stored at room temperature or in the refrigerator confirming other studies on various date cultivars (Shaheen, 1986, Ateyyeh, 2012). Several factors (intrinsic and extrinsic) affect the viability of pollen such as genetic and morphological variability among species, growing conditions of mother plants and methods of their collection and postharvest handling (Barnabás and Kovács, 1997). According to Stanley and Linskens (1974), pollen viability is often higher in most plant species with relative humidity below 60%. Al-Helal (1995) has confirmed that frozen date palm pollen was more viable than refrigerated ones for the long storage periods. According to Mortazavi et al., 2010, Ateyyeh, 2012, the cryopreserved date palm pollen exhibited high viability and germinability with storage at cost medium and long duration. It has been reported that pollen can continue developing during storage and gradually transform into mature and viable pollen (Mortazavi et al., 2010). In the field experiment, fresh pollen gave the highest fruit set and yield per bunch since it possesses the maximum germinability followed by those stored at −30 °C and 4 °C, in contrast to those stored at 28 °C for 12 months. Likewise, the higher enzymatic activity during handling and storage of pollens at ambient temperature could be at the origin of hydrolysis reactions of sugars compared to cold storage or freezing (Yao et al., 2010). Thus, it is necessary to reduce the moisture content in pollen as much as possible (less than 6%) before storage to control chemical reactions in pollen (Verdeil and Pannetier, 1990). In our study, fruit set percentage in field experiment comply with the *in vitro* germination percentage of pollen during storage. These results are consistent with those of Digonnet-Kerhoas and Gay (1990), who showed that *in vitro* germination percentage of pollens would be a fertility index. The decrease in stored pollens fertilization efficiency could also be explained by the decrease in endogenous carbohydrate content that would disturb the emission and lengthening of the pollen tube and/or by disturbances in the pollen-stigma recognition phenomena, as a consequence of the loss of glycoproteins that permeate exine (Charrier, 1990). The pollination period following spathe-cracking might extend over 40 days and greatly vary upon cultivar and climatic conditions especially temperature (El Fawal, 1972). According to Munier (1973), the pollination period can be longer, depends mainly on temperature and possibly extend up to 50 days under lower mean daily temperature. In the Northern hemisphere, pollination takes place from February to April, while it extends from July to early October in the Southern hemisphere. Indeed, calm, dry and warm enough weather is required for successful pollination of date palm (Pereau, 1958, Kadri et al., 2019). In general, the length of the receptivity period of pistillate flowers can vary up to 8 or 10 days depending on the date palm cultivar (Pereau, 1958, Shaheen et al., 1986). Our results showed that pollination performed on the same day of spathe cracking produced the highest fruit set percentage but it decreases to 50% and 90% after 12 and 30 days, respectively. Accordingly, conducting pollination until 6 days following spathe cracking produce fruit set around 67% and thus it is imperative to pollinate within these days. It has been reported that the receptivity period of North African cultivars varies from one cultivar to another (30 days for ‘Bousthami Noire’, 7 days for ‘Deglet Nour’, 8 days for ‘Jihel’ and ‘Ghars’ and only 3 days for ‘Medjhool’, ‘Boufeggous’ and ‘Iklane’) and beyond these limits, parthenocarpic fruit formation are greater than 50% (Djerbi, 1995). In Iraq, Dowson (1982) shown that the receptivity of ‘Ashrasi’ cultivar proved optimal before the natural female spathe cracking, while in ‘Barban’ cultivar it extended up to 20 days after spathe cracking. Our results comply with Ream and Furr (1969), where the female flowers of ‘Deglet Nour’ cultivar retain their receptivity up to 6 days or more after the spathe has cracked and pollination after 15 or 18 days resulted in a moderate or considerable reduction in fruit set, respectively. Indeed, some farmers intended to delay pollination until a few days later to reduce fruit set and consequently crop load for obtaining larger fruit size (Kadri et al., 2019). According to Ream and Furr, 1969, Djerbi, 1995, the maximum receptivity period should not exceed 10 days for the ‘Deglet Nour’ cultivar and 7 days for the ‘Ghars’ cultivar. Concerning the optimal hours of daytime pollination, our results indicated that the highest fruit set percentage and yield were obtained with pollination being performed between 12.0 pm and 15.0 pm. However, pollinations conducted at 8.0 to 11.0 or at the end of the daytime at 16.0 to 17.0 gave the lowest fruit set percentage and yield per bunch, which comply with Dowson, 1982, Djerbi, 1995. Similar results were also found by Iqbal et al. (2014) in which the maximum fruit set and yield were obtained with pollination being performed between 12.0 pm and 1.0 pm. This result is possibly due to climatic conditions where temperature at maximum with minimum relative humidity around that time of the day which might favor pollen germination and fertilization processes.

## 5 Conclusion

This study showed the importance of storage temperature for keeping viability and germinability of date palm pollen. The highest yield per bunch was obtained by using fresh pollen (collected 3 days before pollination) followed by those stored in the freezer (-30 °C) and in the refrigerator (4 °C) while, the lowest yield per bunch obtained with pollen stored at ambient temperature (28 °C). Pollination should be performed between 12.0 pm and 15.0 pm and no later than 6 days following spathe cracking to guarantee acceptable commercial fruit set percentage and yield of ‘Deglet Nour’ date palm cultivar.

## Declaration of Competing Interest

The authors declare that they have no known competing financial interests or personal relationships that could have appeared to influence the work reported in this paper.
